# Novel protein biomarkers for pneumonia and acute exacerbations in COPD: a pilot study

**DOI:** 10.3389/fmed.2023.1180746

**Published:** 2023-06-05

**Authors:** Anna Lena Jung, Maria Han, Kathrin Griss, Wilhelm Bertrams, Christoph Nell, Timm Greulich, Andreas Klemmer, Hendrik Pott, Dominik Heider, Claus F. Vogelmeier, Stefan Hippenstiel, Norbert Suttorp, Bernd Schmeck

**Affiliations:** ^1^Institute for Lung Research, Universities of Giessen and Marburg Lung Center, Philipps-University Marburg, German Center for Lung Research (DZL), Marburg, Germany; ^2^Core Facility Flow Cytometry – Bacterial Vesicles, Philipps-University Marburg, Marburg, Germany; ^3^Medizinische Klinik m.S. Hämatologie und Onkologie, Charité – Universitätsmedizin Berlin, Berlin, Germany; ^4^Department of Internal Medicine/Infectious Diseases and Respiratory Medicine, Charité – Universitätsmedizin Berlin, Corporate Member of Freie Universität Berlin, Humboldt-Universität zu Berlin, and Berlin Institute of Health, Berlin, Germany; ^5^Department of Medicine, Pulmonary and Critical Care Medicine, University Medical Center Marburg, Philipps-University Marburg, German Center for Lung Research (DZL), Marburg, Germany; ^6^Department of Mathematics and Computer Science, Philipps-University Marburg, Marburg, Germany; ^7^Center for Synthetic Microbiology (Synmikro), Philipps-University Marburg, Marburg, Germany; ^8^Member of the German Center of Infectious Disease Research, Marburg, Germany

**Keywords:** pneumonia, COPD, acute exacerbation, severity, biomarker, inflammation

## Abstract

**Introduction:**

Community-acquired pneumonia (CAP) and acute exacerbations of chronic obstructive pulmonary disease (AECOPD) result in high morbidity, mortality, and socio-economic burden. The usage of easily accessible biomarkers informing on disease entity, severity, prognosis, and pathophysiological endotypes is limited in clinical practice. Here, we have analyzed selected plasma markers for their value in differential diagnosis and severity grading in a clinical cohort.

**Methods:**

A pilot cohort of hospitalized patients suffering from CAP (*n* = 27), AECOPD (*n* = 10), and healthy subjects (*n* = 22) were characterized clinically. Clinical scores (PSI, CURB, CRB65, GOLD I-IV, and GOLD ABCD) were obtained, and interleukin-6 (IL-6), interleukin-8 (IL-8), interleukin-2-receptor (IL-2R), lipopolysaccharide-binding protein (LBP), resistin, thrombospondin-1 (TSP-1), lactotransferrin (LTF), neutrophil gelatinase-associated lipocalin (NGAL), neutrophil-elastase-2 (ELA2), hepatocyte growth factor (HGF), soluble Fas (sFas), as well as TNF-related apoptosis-inducing ligand (TRAIL) were measured in plasma.

**Results:**

In CAP patients and healthy volunteers, we found significantly different levels of ELA2, HGF, IL-2R, IL-6, IL-8, LBP, resistin, LTF, and TRAIL. The panel of LBP, sFas, and TRAIL could discriminate between uncomplicated and severe CAP. AECOPD patients showed significantly different levels of LTF and TRAIL compared to healthy subjects. Ensemble feature selection revealed that CAP and AECOPD can be discriminated by IL-6, resistin, together with IL-2R. These factors even allow the differentiation between COPD patients suffering from an exacerbation or pneumonia.

**Discussion:**

Taken together, we identified immune mediators in patient plasma that provide information on differential diagnosis and disease severity and can therefore serve as biomarkers. Further studies are required for validation in bigger cohorts.

## 1. Introduction

Community-acquired pneumonia (CAP) constitutes a crucial cause of morbidity and mortality worldwide, predominantly affecting young children and the elderly (1). Representing the most frequent cause of death due to infection in Europe, CAP accounts for 1 million hospitalizations per year, resulting in 230,000 deaths and socio-economical costs of 10.1 billion € (2–4). Due to the abrupt onset of severe illness, the difficulty in identifying the liable pathogen and the challenge of distinguishing CAP from other acute airway infections, there is an urgent need for easily accessible but specific biomarkers allowing rapid and reliable diagnosis and risk stratification. To date, the diagnosis of CAP mostly relies on chest radiographs and analyses of blood and sputum parameters, as well as auscultation of the lungs and measurement of blood pressure, breathing rate, and recent mental confusion (5). For risk stratification, calculation of clinical scores like CRB65, which are based on defined parameters, roughly allows classification into uncomplicated, moderate, or severe CAP. However, specific biomarkers are still missing (6).

Chronic obstructive pulmonary disease (COPD) represents another highly relevant respiratory disease, being the fourth leading cause of death worldwide with an increasing tendency (7, 8). In Europe, COPD affects about 2–10% of the population and accounts for 3% of all deaths (9). Acute exacerbations of COPD (AECOPD), predominantly caused by viral or bacterial infections, contribute considerably to the burden of the disease. In the United States, AECOPD accounted for 726,000 hospitalizations in 2000, resulting in 119,000 deaths (10) and incurring costs of 30 billion $ (8). Diagnosis of AECOPD mostly relies on increasing cough, sputum, and dyspnoea (11), supported by chest radiographs, blood, and microbiological sputum analyses. Depending on symptoms, AECOPD can be subdivided into type I patients suffering from increasing dyspnoea and optionally sputum, and type II patients with purulent sputum (12). The exacerbation history seems to be the most dependable predictor for future exacerbations (13). As in pneumonia, the availability of reliable biomarkers clearly defining either the entity, stage, or severity of the disease or predicting its progression are of utmost importance.

Biomarkers are classically defined as objectively quantifiable molecules specifically indicating a biological state, which can be physiological or pathogenic, or a response to an expose or (therapeutic) intervention (14). Compared to symptom-based clinical findings, biomarkers have the advantage of allowing a better standardization of measurement and a less investigator-dependent interpretation (15). Due to the variety of molecules, the established handling, and the simplicity of collection, blood is a preferred medium for biomarker analyses (16). At present, the level of C-reactive protein (CRP), the blood sedimentation rate, as well as the leucocyte count, constitute clinical parameters classically indicating inflammation. However, whether this inflammation is of infectious etiology cannot be ascertained (15). Procalcitonin (PCT) represents a current biomarker allowing a more specific discrimination between inflammation of bacterial or non-bacterial origin (17). A potential predictor for mortality in COPD patients is the combination of midrange proadrenomedullin (MR-proADM) and ADO (age, dyspnoea, airflow obstruction), BOD (body mass index, airflow obstruction, dyspnoea) or BODE (body mass index, airflow obstruction, dyspnoea, exercise capability) indices (18, 19). Moreover, MR-proADM could be associated with severe AECOPD but not with CAP (20).

Here, we analyzed selected plasma markers that have been shown to indicate their value as biomarkers for the diagnosis and assessment of the severity of CAP and AECOPD.

## 2. Methods

### 2.1. Patients and controls

Patients with CAP or AECOPD were recruited within 24 h after hospitalization along with healthy controls. Inclusion criteria for patients with CAP included pulmonary infiltrates on chest radiograph and clinical presentation. The inclusion criteria for patients with AECOPD were patients with a previously confirmed diagnosis of COPD and an acute worsening without pulmonary infiltrates on chest radiograph. Patients receiving specific immunosuppressive therapy were excluded from the study, as were pregnant patients and those with human immunodeficiency virus. The BioInflame study was approved by the ethics committee of the Charité – Universitätsmedizin Berlin (no. EA2/030/09) and the University Medical Center Marburg (no. 55/17). All donors were ≥ 18 years of age and provided written informed consent for the use of their blood samples for scientific purposes. Blood plasma was isolated by centrifugation (3,000× *g*; 10 min at room temperature) of one collected Vacutainer ethylenediaminetetraacetic acid tube. After centrifugation, the plasma phase was transferred and stored at −80°C.

### 2.2. Biomarker measurements

Biomarker measurements were performed in duplicate on stored frozen plasma samples from the day of study enrollment. Plasma levels of IL-6, LBP, and IL-2R were measured using the IMMULITE immunoassay system (Siemens Medical Solutions Diagnostics, Germany). Additionally, plasma levels of ELA2, HGF, IL-6, IL-8, LTF, NGAL, resistin, sFas, TSP-1, and TRAIL were measured with the MILLIPLEX^®^ MAP Human Circulating Cancer Biomarker Magnetic Bead Panel 1 and the MILLIPLEX^®^ MAP Human Sepsis Magnetic Bead Panel 3 (Merck Millipore, Germany) on a Luminex MAGPIX^®^ following the manufacturer’s instructions (Luminex Multiplexing Instrument, Merck Millipore). Biomarker levels below the lowest standard were extrapolated corresponding to the equation of the standard curve.

### 2.3. Statistics

Statistical analyses were performed using GraphPad Prism version 9.5 (GraphPad software, Inc., CA, United States). Data were analyzed with non-parametric tests. For comparison of two groups, Mann–Whitney U Test was used. If all three groups were compared, Kruskal-Wallis Test followed by a *post hoc* test (Mann–Whitney U) was used. Receiver Operating Characteristic (ROC) curves were generated to identify biomarkers that clearly distinguish between two groups. To identify a group of parameters that clearly distinguishes between two groups, ensemble feature selection (EFS) was performed.

### 2.4. Ensemble feature selection

Importance analysis of the plasma biomarkers and their ranking was performed using EFS at the web-interface^1^ (21, 22).

### 2.5. Patients and public involvement

Patients and the public were not involved in this study’s design, recruitment and conduct.

## 3. Results

### 3.1. Patient characteristics

We included 27 patients hospitalized with CAP on a regular ward, ten patients with an acute exacerbation of COPD, and 22 healthy controls (Table 1). Samples were taken from all the patients within the first 24 h after hospital admission. The healthy control group was significantly younger than the CAP group, but CAP and AECOPD did not differ significantly. It also contained more female subjects, whereas in the CAP group, men were predominant, and the AECOPD group was balanced in terms of gender. In CAP patients, intermediate severity scores were most frequent, but mild or severe cases were also present (Table 2). All patients underwent routine microbiological testing, but did not show bacterial growth in blood culture and no specific causative pathogen in sputum culture. The majority of CAP patients (*n* = 17) were prescribed beta-lactam antibiotics, either alone or in combination with a beta-lactamase inhibitor or macrolide. Additionally, five patients received cephalosporine antibiotics, and four patients were prescribed fluoroquinolone antibiotics. In the AECOPD group, most patients experienced at least one exacerbation per year. They showed higher levels of airflow obstruction and breathlessness (Table 3). For AECOPD patients, systemic corticosteroids (oral or intravenous) were administered as the first-line treatment upon hospital admission, followed by antibiotics (amoxicillin/clavulanic acid). Four out of the ten AECOPD patients were already under systemic corticosteroid treatment prior to hospital admission. As COPD patients are more likely to develop pneumonia and suffer from more severe pneumonia (23), the CAP group was subdivided into patients with underlying COPD (*n* = 8) and without COPD (*n* = 19) (Table 4). All of the patients included here were effectively treated with appropriate medical care, including antibiotics and supportive measures, and were able to overcome the infection and recover; none of them died.

**Table 1:** General characteristics of healthy controls and patients with CAP or AECOPD.

	Healthy controls (*N* = 22)	CAP (*N* = 27)	AECOPD (*N* = 10)
Mean age [years ± SD]	41.0 ± 11.5	64.3 ± 17.7	62.4 ± 9.2
Gender m/f (%)	8/14 (36.4/63.6)	16/11 (59.2/40.8)	5/5 (50/50)

Data are presented as mean ± SD or *n* (%). CAP, community-acquired pneumonia; AECOPD, acute exacerbations of COPD.

**Table 2:** Clinical characteristics of patients suffering from CAP.

CRB65 (%)		CURB (%)		PSI class (%)	
0	4 (15)	0	4 (15)	1	5 (19)
1	15 (56)	1	10 (37)	2	1 (4)
2	6 (22)	2	10 (37)	3	4 (15)
3	2 (7)	3	2 (7)	4	12 (44)
4	0 (0)	4	1 (4)	5	5 (19)
sO_2_ ± SD [%]		94.5 ± 3.1			
Body temperature ± SD [°C]		37.4 ± 1.2			
CRP ± SD [mg/dL]		14.0 ± 11.0			
WBC ± SD [1/nL]		10.4 ± 4.5			

Data are presented as mean ± SD or *n* (%). CRB65, confusion, respiratory rate, blood pressure, 65 years of age, and older; CURB, confusion, urea nitrogen, respiratory rate, and blood pressure; PSI, pneumonia severity index; CRP, C-reactive protein; WBC, white blood cell count.

**Table 3:** Clinical characteristics of patients suffering from AECOPD.

GOLD stage (%)		GOLD group (%)		mMRC (%)	
I	0 (0)	A	0 (0)	I	1 (10)
II	2 (20)	B	0 (0)	II	3 (30)
III	0 (0)	C	2 (20)	III	6 (60)
IV	6 (60)	D	6 (60)	IV	0 (0)
ND	2 (20)	ND	2 (20)		
Exacerbation/year ± SD		1.2 ± 0.7			
Pack years ± SD		62 ± 47			
6MWT ± SD [m]		225 ± 131			
CRP ± SD [mg/dL]		2.4 ± 1.9			
WBC ± SD [1/nL]		10.6 ± 2.4			

Data are presented as mean ± SD or *n* (%). GOLD, global initiative for chronic obstructive lung disease; mMRC, modified British Medical Research Council; 6MWT, six-minutes walking test; CRP, C-reactive protein; WBC, white blood cell count.

**Table 4:** Clinical characteristics of CAP patients with and without COPD.

		CAP + COPD (*n* = 8)	CAP − COPD (*n* = 19)
GOLD (%)	I	0 (0)	
	II	2 (25)	
	III	1 (12.5)	
	IV	1 (12.5)	
	Not classified	4 (50)	

PSI risk class (%)	1	0 (0)	5 (26.3)
	2	0 (0)	1 (5.3)
	3	2 (25)	2 (10.5)
	4	5 (62.5)	7 (36.8)
	5	1 (12.5)	4 (21.1)

Mean age [years ± SD]		72.9 ± 7.5	60.7 ± 19.6
Gender m/f (%)		5/3 (62.5/37.5)	11/8 (57.9/42.1)

Data are presented as mean ± SD or *n* (%). GOLD, global initiative for chronic obstructive lung disease; PSI, pneumonia severity index.

### 3.2. Differential abundance of biomarkers in plasma

**Figure 1:**
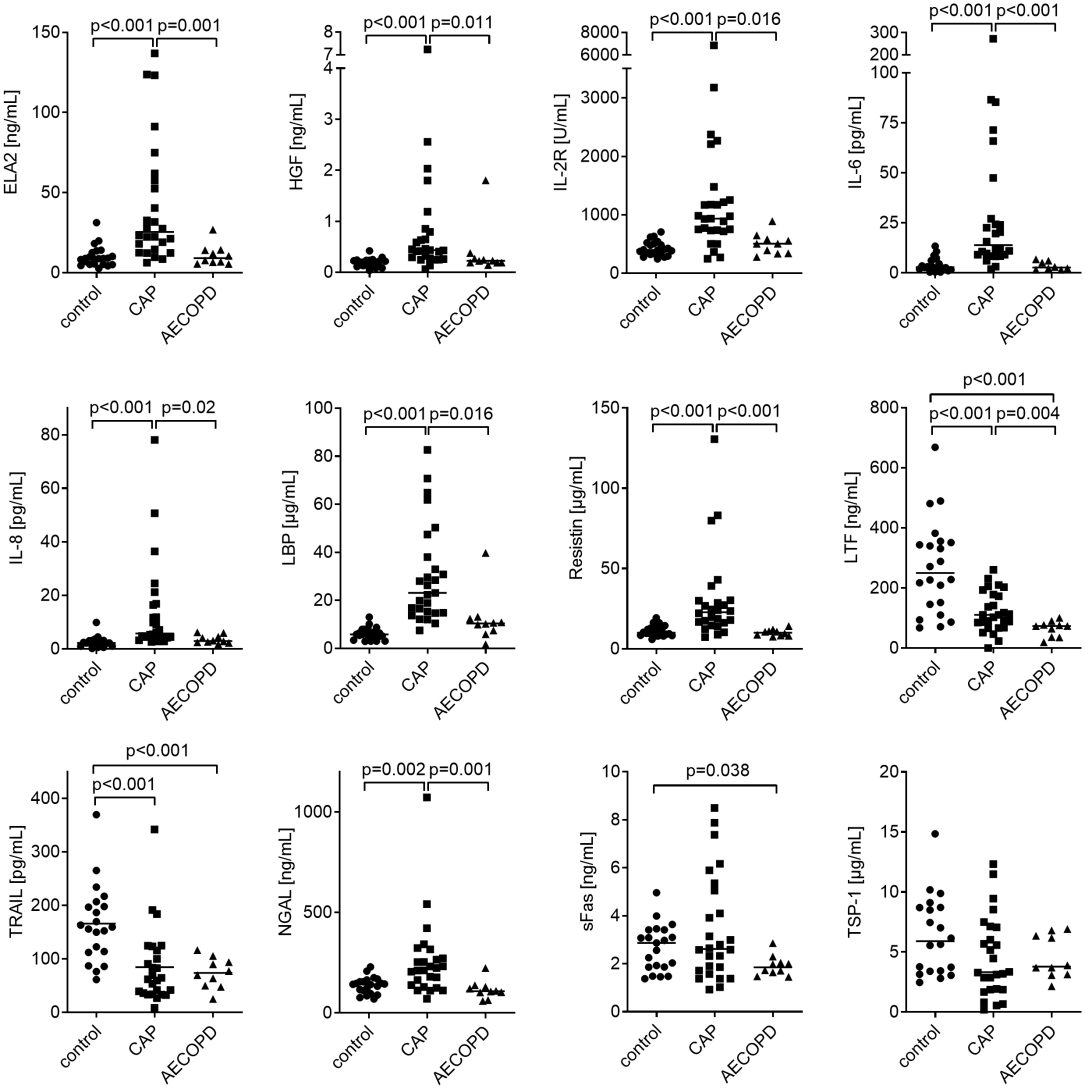
Biomarker expression in plasma samples from CAP and AECOPD patients compared to healthy controls. Data are presented as scatter plots with the median shown as a line. Statistics: Kruskal-Wallis test was performed and *p* ≤ 0.001 was considered significant.

We performed multiplex analysis of molecules associated with sepsis and tumor diseases to test their significance in plasma samples as biomarkers for the diagnosis of CAP and AECOPD. The individual statistical analysis showed that eight of the twelve analyzed potential biomarkers were differentially expressed in CAP compared to healthy controls. Plasma levels of ELA2, HGF, IL-2R, IL-6, IL-8, LBP, and resistin as well as leukocyte, monocyte, and neutrophil counts were significantly elevated (Figure 1; Supplementary Figure S1). NGAL showed the same trend (*p* = 0.002). In contrast, levels of LTF and TRAIL were significantly reduced. No significant differences were found for sFas and TSP-1. In AECOPD patients, leukocyte, monocyte, and neutrophil counts were significantly higher compared to healthy controls (Figure 1; Supplementary Figure S1). Levels of LTF and TRAIL were significantly lower. sFas was reduced as well (*p* = 0.038). Comparing CAP with AECOPD, patients with CAP revealed significantly higher levels of ELA-2, IL-6, resistin, NGAL and CRP. To test whether some biomarkers might be expressed simultaneously, we performed Pearson’s correlation analysis among all significantly expressed proteins and found the best positive correlations between resistin with NGAL and IL-6 with IL-8 (Supplementary Figure S2).

### 3.3. Diagnostic value

**Figure 2:**
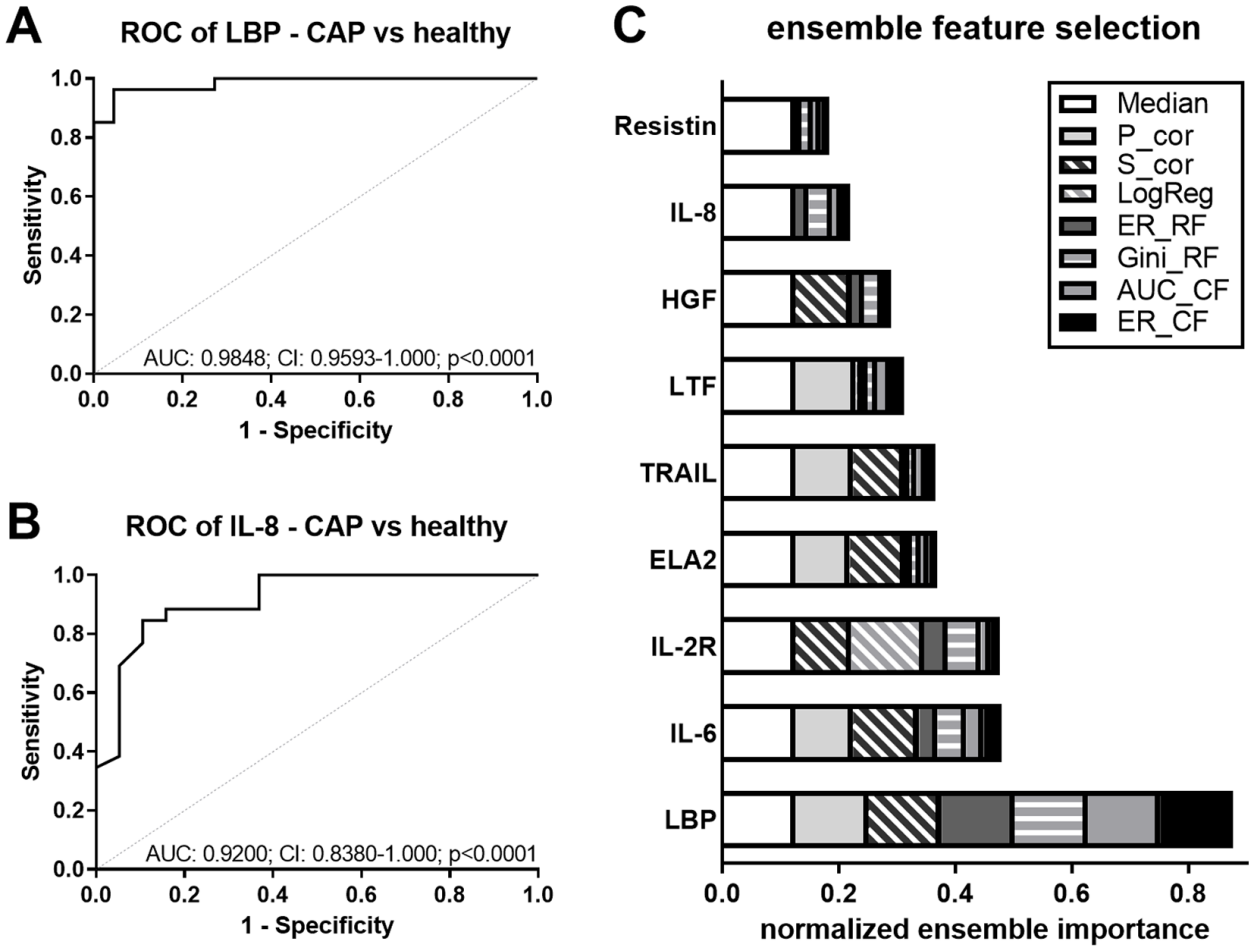
Discrimination between CAP and healthy controls. **(A,B)** ROC curves for the discrimination between healthy controls and CAP using LBP **(A)** and IL-8 **(B)**. Dashed line (grey) shows line of identity. AUC, area under the curve; CI, confidence interval, and *p*-values are depicted in the graphs. **(C)** Ensemble feature selection for healthy controls and CAP using all significantly regulated biomarkers. The cumulative barplot shows individual features for all feature selection methods. P_cor, Pearson product moment correlation; S_cor, Spearman’s rank correlation; LogReg, logistic regression; ER_RF, error-rate-based variable importance measure embedded in *randomForest*; Gini_RF, Gini-index-based variable importance measure embedded in *randomForest*; AUC_CF, area under the curve embedded in *cforest*; ER_CF, error-rate-based variable importance measure embedded in *cforest*.

To augment the diagnostic significance of the analyzed parameters and statistically link each parameter to the prediction of CAP or AECOPD, respectively, we calculated the area under the curve (AUC) of the receiver operating characteristics (ROC) curve for all significantly regulated biomarkers from Figure 1. In CAP patients compared to healthy controls, the AUC values of LBP, IL-8, IL-6, HGF, IL-2R, ELA2, resistin, TRAIL, and LTF had significant *p*-values, but only the top two (LBP and IL-8) are presented in Figure 2. As the diagnostic potential of a composite of potential biomarkers might be higher than single parameters, feature selection was performed. To this end, EFS was chosen, as it combines eight feature selection methods and thereby improves the prediction performance (21). This multivariate analysis returns the importance of each significantly regulated marker, which are normalized quantifications of the predictive capabilities of the given variables (Figure 2C). The selected features are LBP, IL-6, and IL-2R, which we define as a marker panel for CAP diagnosis.

As it is critical in clinical routine to determine which patient has a higher risk to develop a more severe form of pneumonia, we aimed to find plasma markers for this discrimination. We subdivided the CAP patients according to the pneumonia severity index (PSI) risk class (uncomplicated (u) CAP: 1–3; severe (s) CAP: 4–5) and tested whether our collected parameters support this stratification. Smaller group sizes after separation (uCAP: 10; sCAP: 17) precluded robust statistical differentiation between the two groups, but TRAIL emerged as the most appropriate parameter allowing discrimination of uCAP and sCAP with a value of *p* < 0.05 (Supplementary Figure S3A). In ROC analysis, TRAIL had an AUC of 0.7406 (Supplementary Figure S3B). To combine the power of all measured plasma parameters, EFS was performed. The selected features were LBP, sFas, TRAIL, IL-6, and IL-8, of which LBP ranked highest (0.8) according to its normalized ensemble importance (Supplementary Figure S3C).

To elucidate the diagnostic value of the differentially expressed markers in AECOPD plasma compared to healthy controls, ROC analysis was performed for the significantly regulated biomarkers from Figure 1. AUC values of LTF and TRAIL were > 0.89 with significant *p*-values (Supplementary Figure S4).

**Figure 3:**
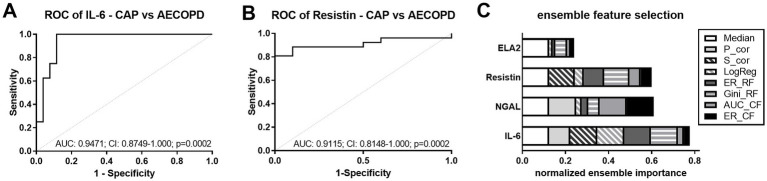
Discrimination between CAP and AECOPD. **(A,B)** ROC curves for the discrimination between CAP and AECOPD by IL-6 **(A)** and resistin **(B)**. Dashed line (grey) shows line of identity. AUC, area under the curve; CI, confidence interval and *p*-values are depicted in the graphs. **(C)** EFS for CAP and AECOPD for all significantly regulated biomarkers. The cumulative barplot shows individual features for all feature selection methods. P_cor, Pearson product moment correlation; S_cor, Spearman’s rank correlation; LogReg, logistic regression; ER_RF, error-rate-based variable importance measure embedded in *randomForest*; Gini_RF, Gini-index-based variable importance measure embedded in *randomForest*; AUC_CF, area under the curve embedded in *cforest*; ER_CF, error-rate-based variable importance measure embedded in *cforest*.

As CAP and AECOPD often exhibit similar symptoms and can be difficult to distinguish, we performed ROC analyses comparing AECOPD and CAP patients based on all significantly regulated plasma proteins from Figure 1. AUC values of IL-6, resistin, NGAL, and ELA2 showed significant *p*-values, but only the top two (IL-6 and resistin) are presented in Figures 3A,B. Multi-parametric analysis was performed to discriminate CAP from AECOPD. The selected features were IL-6, NGAL, and resistin, of which IL-6 ranked highest (0.78) according to its normalized ensemble importance (Figure 3C).

**Figure 4:**
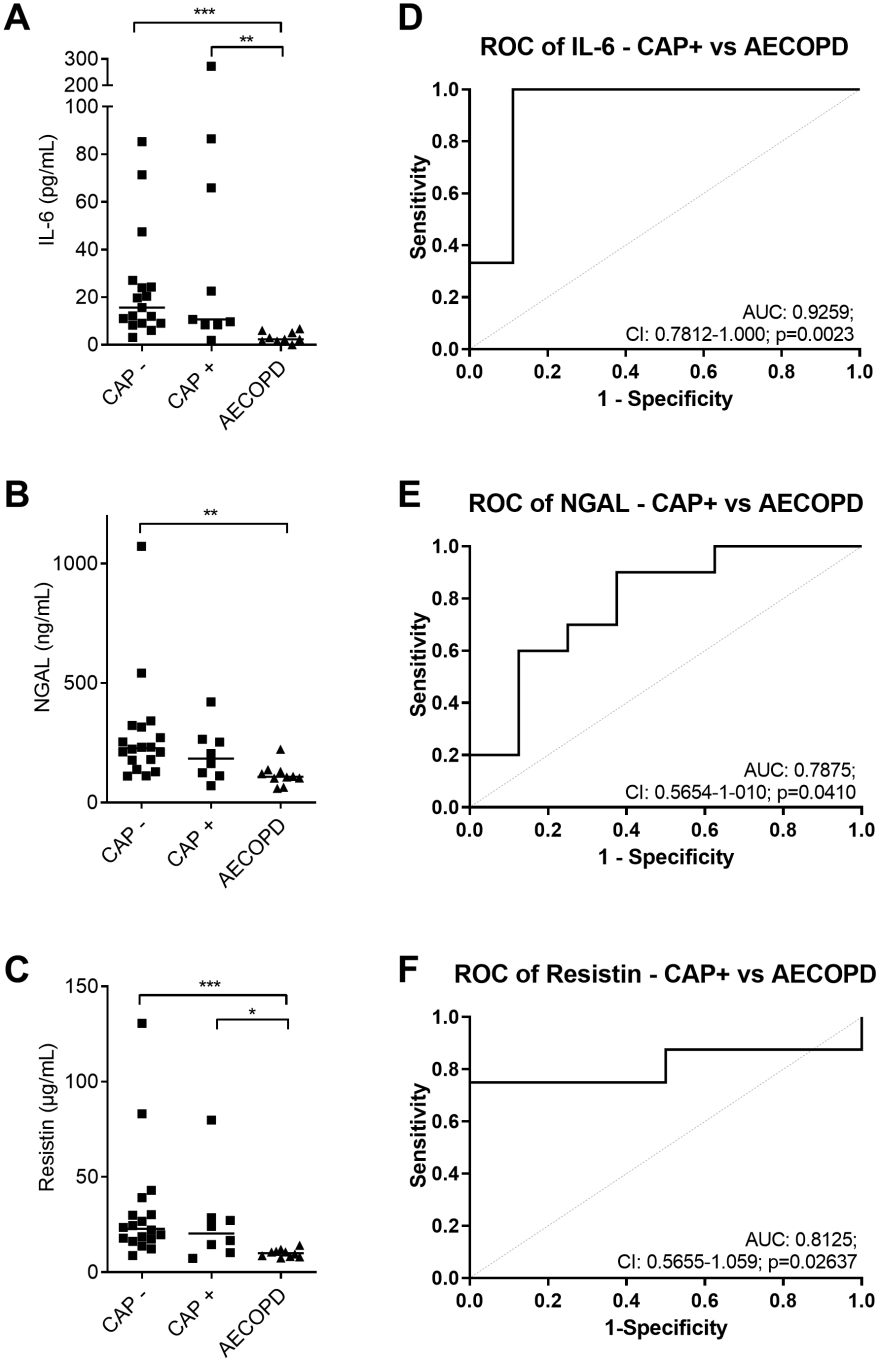
Discrimination between AECOPD and CAP with pre-existing COPD. **(A–C)** Amount of IL-6 **(A)**, NGAL **(B)**, and resistin **(C)** in plasma samples from CAP (−/+ COPD) and AECOPD patients. Data are presented as scatter plots and the line shows the median. **(D–F)** ROC curves for the discrimination between CAP+COPD and AECOPD by IL-6 **(D)**, NGAL **(E)**, and resistin **(F)**. Dashed line (grey) shows line of identity. AUC, area under the curve; CI, confidence interval (CI), and *p*-value are depicted in the graph. Statistics: Kruskal-Wallis test was performed and *p* < 0.05 was considered significant (*p < 0.05, **p < 0.01, ***p < 0.001).

The selected features to discriminate CAP and AECOPD from the EFS analysis (Figure 3C) were again tested for their reliability when CAP patients with and without pre-existing COPD were compared to AECOPD patients. IL-6 and resistin were still significantly different when the CAP patient had a pre-existing COPD (Figures 4A–C). The ROC analyses for IL-6, NGAL, and resistin, between AECOPD and CAP+COPD revealed AUCs >0.79 with significant *p*-values (Figures 4D–F).

## 4. Discussion

In this study, we investigated a set of plasma proteins as potential biomarkers for CAP or AECOPD, respectively.

In CAP patients, we found that the plasma levels of ELA2, HGF, IL-2R, IL-6, IL-8, LBP, and resistin were elevated, while LTF and TRAIL were reduced. Among these, LBP, IL-6, and IL-2R are features for CAP diagnosis, and the combination of LBP, sFas, TRAIL, IL-6, and IL-8 allowed discrimination between uncomplicated and severe forms of pneumonia.

LBP is known to be involved in the acute-phase response against gram-negative bacteria by binding the lipid A moiety of soluble LPS and presenting it to CD14 on cellular membranes. It is mainly produced in the liver and its plasma concentration increases exponentially during acute inflammatory response (24), but it is also found in alveolar fluid (25). It was found that the serum level of LBP was significantly higher in patients with sCAP compared to uCAP and correlated with disease severity, which we also observed. Moreover, LBP has a higher predictive power than CRB-65 (26). Tejera et al. found higher serum levels of LBP in hospitalized CAP patients (27). Likewise, the pro-inflammatory cytokine IL-6 has been found to be increased in CAP patients in other studies. The serum levels of IL-6 were significantly higher in high-risk compared to low-risk CAP patients, and its level correlated with disease severity and decreased on day 3 and 5 after hospitalization (28). Bacci et al. and Andrijevic et al. made similar observations, as IL-6 levels correlated with PSI and CURB score and high IL-6 serum levels were associated with higher lethality of hospitalized patients and decreased from the day of admission to day seven of treatment (29, 30). In our study, IL-6 showed a trend of gradual upregulation with pneumonia severity, it was a selected feature for discrimination between uCAP and sCAP and correlated with the plasma levels of IL-8, which has been described to be significantly higher in sCAP patients compared to milder CAP (30). Soluble IL-2R is a marker for T-cell activation (31). sFas, Fas ligand, and their ratio were associated with a high sepsis-related organ failure assessment (SOFA) score in patients with bacteremia (32). We found it to be decreased in CAP compared to healthy controls but higher in sCAP compared to uCAP. TRAIL is a type II transmembrane protein and belongs to the TNF/TNFR superfamily, which is involved in infection control and the regulation of both innate and adaptive immune responses (33). TRAIL level in airway epithelial cells of COPD patients is elevated (34). The serum concentration of TRAIL has been demonstrated to negatively correlate with pulmonary function (35).

In AECOPD patients, LTF, and TRAIL were significantly reduced in plasma samples, and both had AUC values indicating discriminative potential to differentiate between AECOPD and healthy controls. LTF is an iron-binding protein and provides protection against pathogens and their metabolites. Moreover, it has bactericidal properties that lead to the release of LPS from the outer membrane of gram-negative bacteria (36). It has the capability to enhance phagocytosis of pathogens and cell adherence and controls the release of pro-inflammatory cytokines (37, 38). LTF has been described to be down-regulated in asthmatic patients (39) as observed in our CAP and AECOPD patients.

Since the incidence of pneumonia in COPD patients is almost twice as high as in the general population (40), COPD patients can suffer from both AEs and CAP, which require different clinical treatments and must be differentiated accordingly. This differential diagnosis is traditionally based on radiological findings. It is important to have biological markers to improve the differential diagnosis, reduce unnecessary use of antibiotics, and lower mortality and expenditures for care. Comparing CAP and AECOPD patients, CAP patients had significantly higher levels of IL-6 and CRP. Huerta et al. also observed higher IL-6 levels in CAP patients compared to AECOPD as well (41). EFS revealed that IL-6, NGAL, and resistin are suitable markers for discrimination between AECOPD and CAP, and the discrimination was still possible with these selected features even when the CAP patient additionally suffers from COPD. Resistin, which correlated with NGAL, is an important pro-inflammatory cytokine produced by monocytes and epithelial cells (42). The levels of resistin were higher in sCAP patients compared to uCAP patients, which is in line with the observation that sepsis patients also have elevated levels of resistin (43, 44). Several studies indicate that resistin and NGAL are increased in sepsis patients and serve as markers for disease severity (45–47). Plasma NGAL concentrations increased with severity and could predict mortality (48), which we did not test. NGAL and IL-6 were significantly higher in patients with lower respiratory tract infections compared to healthy controls, but Liu et al. did not observe differences between CAP and AECOPD in their study (49).

The proposed biomarkers for CAP diagnosis and differential diagnosis between CAP and AECOPD should be validated in larger cohorts in the future. As we had significantly younger healthy controls compared to CAP patients, we wanted to exclude the possibility that the increase in their plasma expression was solely due to the difference in age between the two groups. Therefore, we tested for correlation between age and the suggested markers, but did not observe any correlation (all *R*^2^ < 0.05; Supplementary Figure S5).

We identified a plasma biomarker panel that shows potential for CAP and AECOPD diagnosis and stratification even in CAP cases with COPD as an underlying disease. Future studies with larger cohorts and a multicentric design are needed for further evaluation.

## Data availability statement

The raw data supporting the conclusions of this article will be made available by the authors, without undue reservation.

## Ethics statement

BioInflame study was approved by the ethics committee of the Charité–Universitätsmedizin Berlin (no. EA2/030/09) and the University Medical Center Marburg (no. 55/17). The patients/participants provided their written informed consent to participate in this study.

## Author contributions

AJ analyzed the data and wrote the manuscript in consultation with WB and BS. KG performed the experiments. CN and DH performed statistical analyses. MH, TG, AK, HP, CV, SH, and NS collected the patient’s material. BS supervised the manuscript. All authors contributed to the article and approved the submitted version.

## Funding

This work has been funded in part by the Bundesministerium für Bildung und Forschung (Federal Ministry of Education and Research: PermedCOPD – FKZ 01EK2203A; ERACoSysMed2 – SysMed-COPD – FKZ 031L0140; e:Med CAPSYS – FKZ 01ZX1604E), the Deutsche Forschungsgemeinschaft (SFB/TR-84 TP C01), and the von-Behring-Röntgen-Stiftung (66-LV07) to BS, the Deutsche Forschungsgemeinschaft (SFB/TR-84 TP A04/B06) to SH, as well as the Hessisches Ministerium für Wissenschaft und Kunst (LOEWE Diffusible Signals LOEWE-Schwerpunkt Diffusible Signals) to DH, AJ, and BS. Open Access funding provided by the Open Access Publishing Fund of Philipps-Universität Marburg with support of the Deutsche Forschungsgemeinschaft (DFG, German Research Foundation).

## Conflict of interest

The authors declare that the research was conducted in the absence of any commercial or financial relationships that could be construed as a potential conflict of interest.

## Publisher’s note

All claims expressed in this article are solely those of the authors and do not necessarily represent those of their affiliated organizations, or those of the publisher, the editors and the reviewers. Any product that may be evaluated in this article, or claim that may be made by its manufacturer, is not guaranteed or endorsed by the publisher.

## Supplementary material

The Supplementary material for this article can be found online at: https://www.frontiersin.org/articles/10.3389/fmed.2023.1180746/full#supplementary-material

## Footnotes

^1^http://efs.heiderlab.de/.
