# Supplementary material for: Novel protein biomarkers for pneumonia and acute exacerbations in COPD: a pilot study
Source: Front Med (Lausanne). 2023 Jun 5;10:1180746. doi: 10.3389/fmed.2023.1180746 (PMC10277477; doi:10.3389/fmed.2023.1180746)
Supplement: Supplementary file 1 [file Data_Sheet_1.docx]

Supplementary Material

Novel protein biomarkers for pneumonia and acute exacerbations in COPD: a pilot study

Anna Lena Jung*, Maria Han, Kathrin Griss, Wilhelm Bertrams, Christoph Nell, Timm Greulich, Andreas Klemmer, Hendrik Pott, Dominik Heider, Claus F. Vogelmeier, Stefan Hippenstiel, Norbert Suttorp, Bernd Schmeck

*** Correspondence:** Anna Lena Jung: anna.jung@uni-marburg.de

# Supplementary Figures

**Figure S1:**


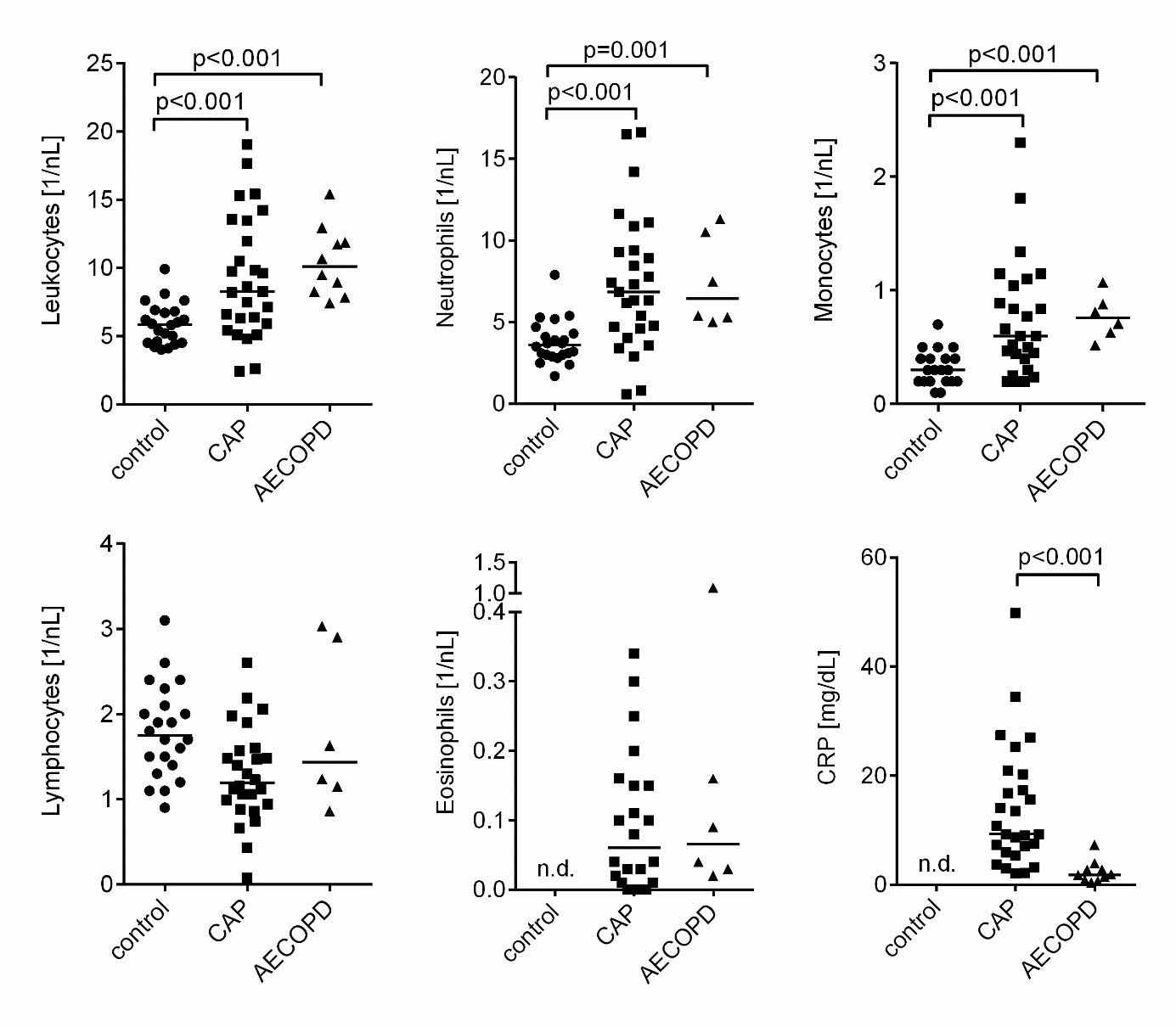


**Figure S1:** Blood cell counts and CRP levels from CAP and AECOPD patients in comparison to healthy controls. Data is presented in scatter plots and the line is showing the median. Statistics: Kruskal-Wallis test was performed to compare all three groups; Mann-Whitney-U test was performed to compare two groups; p≤0.001 was considered to be significant. n.d.: not determined.

**Figure S2:**


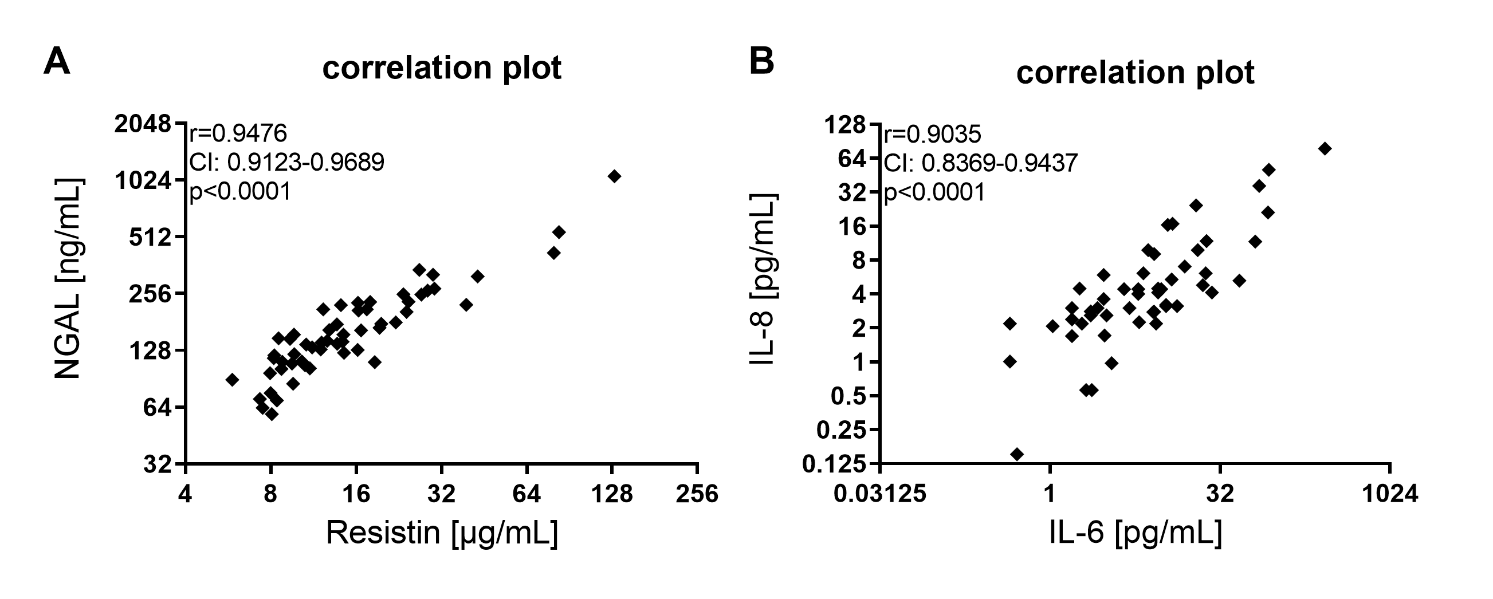


**Figure S2:** Correlation plots of the top two positive correlations. A) correlation plot for NGAL with Resistin. B) correlation plot for IL-8 with IL-6. Pearson’s correlation coefficient (r), 95% confidence interval (CI) and p-value are depicted in the graphs.

**Figure S3:**


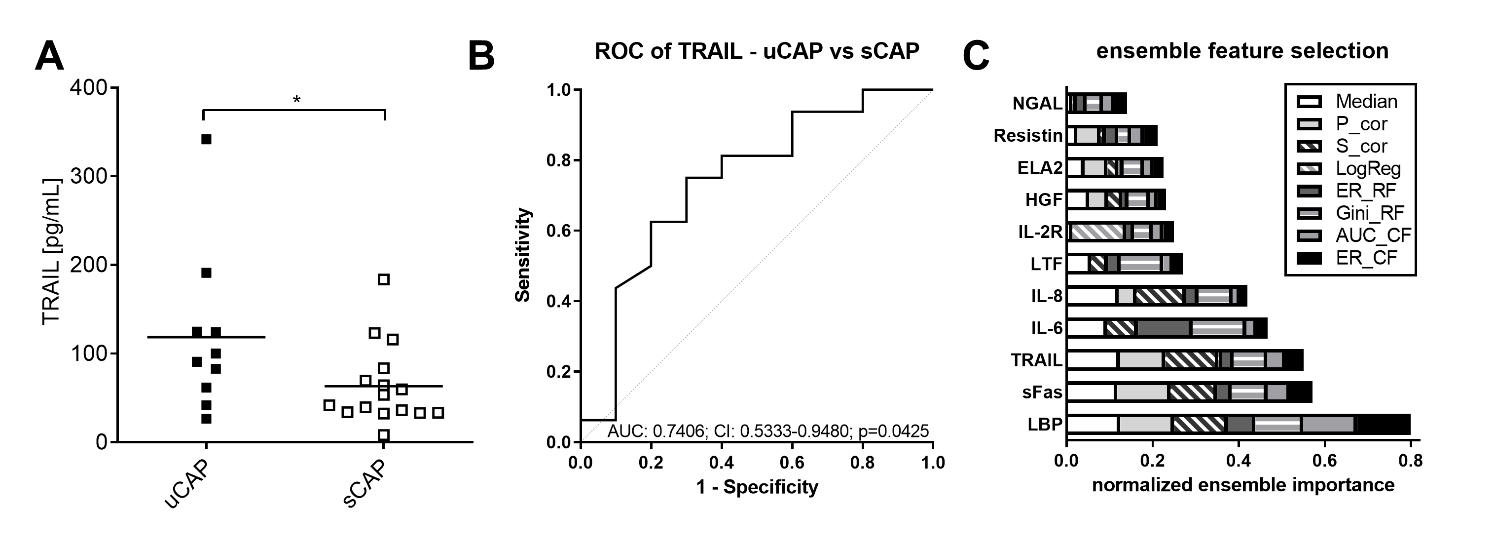


**Figure S3:** CAP severity markers. A) TRAIL expression in plasma samples from uCAP and sCAP patients. Data is presented as scatter plots and the line is showing the median. B) ROC curve for the discrimination between uCAP and sCAP by TRAIL. Dashed line (grey) shows line of identity. Area under the curve (AUC), confidence interval (CI) and p-value are depicted in the graph. C) EFS for uCAP and sCAP. Cumulative barplot of individual features for all feature selection methods is shown. P_cor: Pearson product moment correlation, S_cor: Spearman’s rank correlation, LogReg: logistic regression, ER_RF: error-rate-based variable importance measure embedded in *randomForest*, Gini_RF: Gini-index-based variable importance measure embedded in *randomForest*, AUC_CF: area under the curve embedded in *cforest*, ER_CF: error-rate-based variable importance measure embedded in *cforest*. Statistics: Mann-Whitney U test was performed and p<0.05 was considered to be significant.

**Figure S4:**


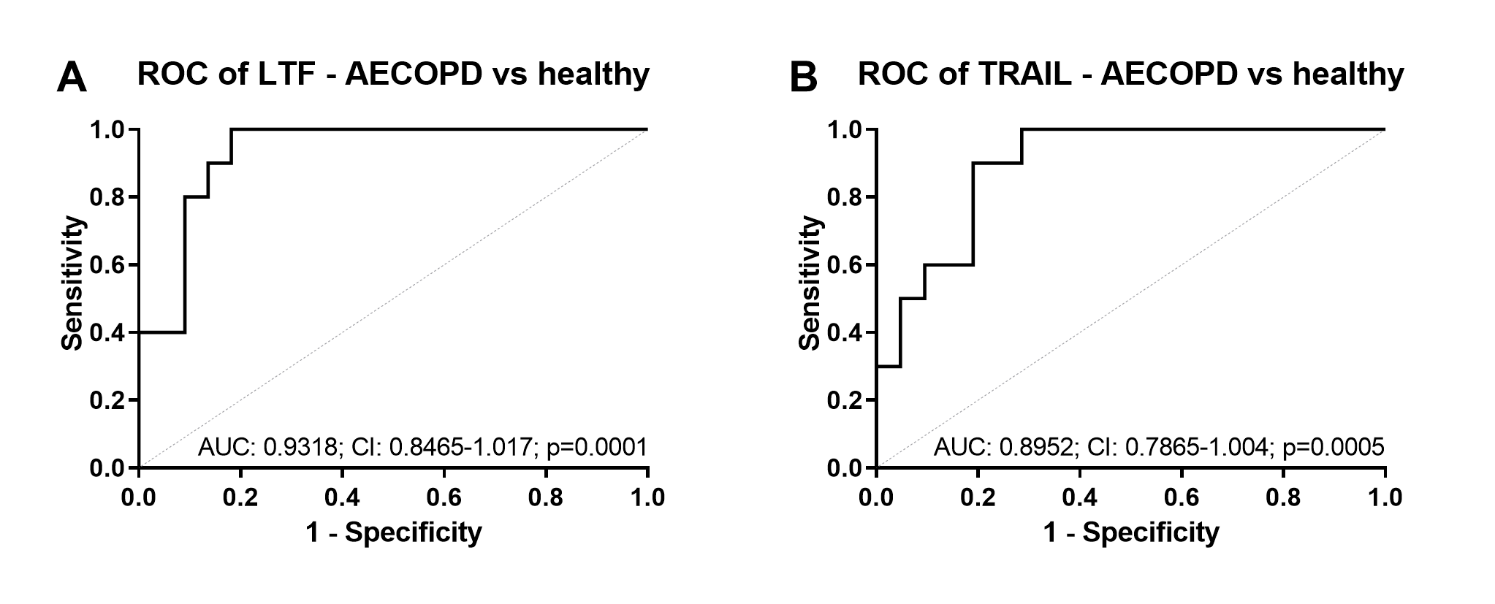


**Figure S4:** Discrimination between AECOPD and healthy. A/B) ROC curves for the discrimination between healthy and AECOPD by LTF (A) and TRAIL (B). Dashed line (grey) shows line of identity. Area under the curve (AUC), confidence interval (CI) and p-values are depicted in the graphs.

**Figure S5:**


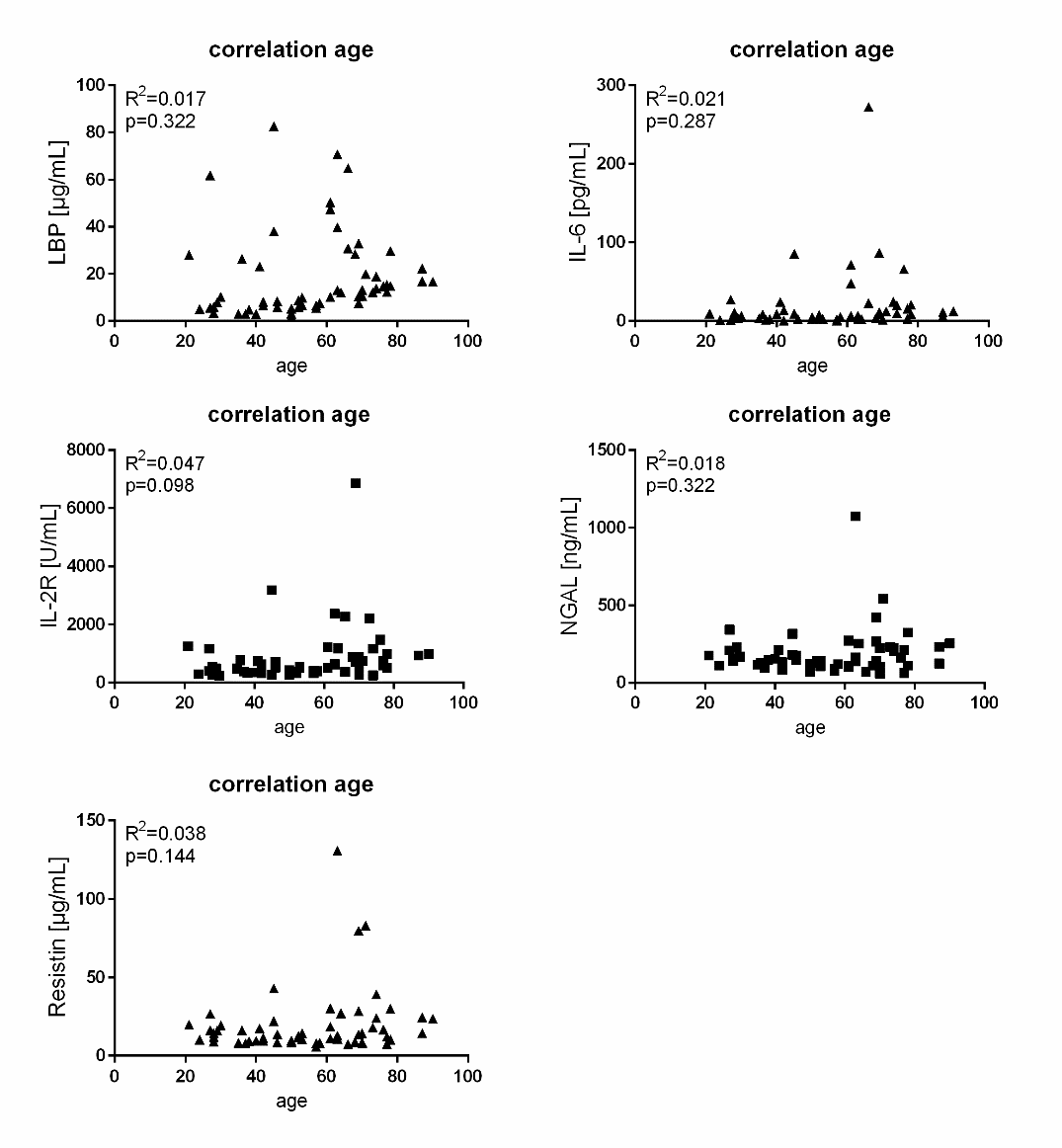


**Figure S5:** Correlation of cytokines with age. Pearson correlation for indicated biomarkers with age was calculated. R^2^ and p-value are depicted in the graph.
